# Quality of life in children with infrequent congenital heart defects: cohort study with one-year of follow-up

**DOI:** 10.1186/s12955-019-1265-z

**Published:** 2020-01-06

**Authors:** Karen Moreno-Medina, Magally Barrera-Castañeda, Catalina Vargas-Acevedo, Alberto E. García-Torres, Miguel Ronderos, Manuel Huertas-Quiñones, Silvana Cabrera, María Teresa Domínguez, Nestor Sandoval Reyes, Rodolfo J. Dennis

**Affiliations:** 1 Research Department, Fundación Cardioinfantil-Instituto de Cardiología, Calle 163 A # 13 B - 60, 110131 Bogotá, Colombia; 2 PINOCCHIO Program, Research Department, Fundación Cardioinfantil-Instituto de Cardiología, Calle 163 A # 13 B - 60, Bogotá, Colombia; 3 Pediatrics Resident, Department of Pediatrics, Universidad de los Andes-HUFSFB, Carrera 7 # 116-5, Bogotá, Colombia; 4 Institute of Congenital Heart Defects, Fundación Cardioinfantil-Instituto de Cardiología, Calle 163 A # 13 B-60, Bogotá, Colombia; 5 Escuela de Medicina y Ciencias de la Salud, Universidad del Rosario, Carrera 24 # 63C-69, Bogotá, Colombia; 6 Pediatrics Department, Universidad Nacional de Colombia, Carrera 45, Bogotá, Colombia; 7 Department of Clinical Psychology, Fundación Cardioinfantil-Instituto de Cardiología, Calle 163 A # 13 B-60, Bogotá, Colombia; 8 Cardiovascular Surgery Department, Fundación Cardioinfantil-Instituto de Cardiología, Calle 163 A # 13 B-60, Bogotá, Colombia

**Keywords:** Congenital heart defects, Quality of life, Caregivers, Colombia

## Abstract

**Background:**

The evidence regarding patient related outcomes in children with infrequent congenital heart defects (I-CHD) is very limited. We sought to measure quality of life (QoL) in children with I-CHD, and secondarily, to describe QoL changes after one-year of follow-up, self-reported by children and through their caregivers’ perspective.

**Methods:**

We assembled a cohort of children diagnosed with an I-CHD in a cardiovascular referral center in Colombia, between August 2016 and September 2018. At baseline and at one-year follow-up, a clinical psychology assessment was performed to establish perception of QoL. The Pediatric Quality of Life Inventory (PedsQL) 4.0 scale was used in both general and cardiac modules for patients and for their caregivers. We used a Mann-Whitney U test to compare scores for general and cardiac modules between patients and caregivers, while a Wilcoxon test was used to compared patients’ and caregivers’ baseline and follow-up scores. Results are presented as median and interquartile range.

**Results:**

To date, QoL evaluation at one-year follow-up has been achieved in 112/157 patients (71%). Self-reported scores in general and cardiac modules were higher than the QoL perceived through their caregivers, both at baseline and after one-year of follow-up. When compared, there was no statistically significant difference in general module scores at baseline between patients (median = 74.4, IQR = 64.1–80.4) and caregivers scores (median = 68.4, IQR = 59.6–83.7), *p* = 0.296. On the contrary, there was a statistical difference in baseline scores in the cardiac module between patients (median = 79.6, IQR = 69.7–87.4) and caregivers (median = 73.6, IQR = 62.6–84.3), *p* = 0.019. At one-year of follow-up, scores for the general module between patients (median = 72.8, IQR = 59.2–85.9) and caregivers (median = 69.9, IQR = 58.1–83.7) were not statistically different (*p* = 0.332). Finally, a significant difference was found for cardiac module scores between patient (median = 75.0, IQR = 67.1–87.1) and caregivers (median = 73.1, IQR = 59.5–83.8), *p* = 0.034.

**Conclusions:**

QoL in children with I-CHD can be compromised. However, children have a better perception of their QoL when compared with their caregivers’ assessments. To provide high-quality care, besides a thorough clinical evaluation, QoL directly elicited by the child should be an essential aspect in the integral management of I-CHD.

## Background

Congenital heart defects (CHD) are the most frequent congenital anomaly at birth, with an incidence of 9/1000 live births [1, 2]. With the development and further evolution of the cardiopulmonary bypass machine, cardiac surgeons along with the entire heart team have been able to correct complex heart defects with increasingly complex surgical and percutaneous procedures [3, 4]. In hand with the improvement in medical and intensive care management, the probability of survival beyond infancy changed from 25% in 1950 to 90% in 2010 [4, 5]. We have passed from a mortality of close to 100% in complex cases to less than 2% in specialized cardiovascular centers [3, 4, 6].

Beyond clinical and hemodynamic improvement, along with better survival rates past the critical period, patients with CHD are reaching adolescence and adulthood; thus, another issue that now concerns the clinical team is their perception of health related quality of life (QoL) [7, 8]. CHD patients constitute a unique group that require special medical care, with emotional and cognitive needs that are a consequence of their condition, which may have an impact on the patient’s and their family’s QoL [4].

Previous studies [7, 9–11] have shown that QoL perception in children with CHD is compromised since they have lower total scores in Pediatric Quality of Life Inventory -PedsQL- (mean = 75.8, SD = 15.9) when compared to healthy children (mean = 86.2, SD = 11.7). The same pattern is observed in adolescence (mean = 79.0, SD = 15.4), when comparing adolescents with CHD with their healthy counterparts (mean = 86.9, DS = 11.8). Perception of QoL is proportional to CHD severity, with lower scores for more severe conditions, especially on physical and psychosocial domains, perceived by both children and parents or caregivers [9, 11]. Additionally, when QoL is assessed through a parent or caregiver, total scores are lower, for both children (mean = 74.7, SD = 16.7) and adolescents (mean = 74.2, SD = 17.8). However, the same is observed when caregivers are asked about the QoL of healthy children (mean = 84.9, SD = 12.9) or adolescents (mean = 85.0, SD = 12.8) [9].

These findings have increased awareness in regard to the importance of including patient-related outcomes (PROs), such as QoL, during patient assessment. When it comes to clinical decision making [12], QoL evaluation should be elicited directly from the patient (or best measured from the patient’s perspective); however, the body of evidence available in this specific area is still limited, especially in children. This is of critical importance, since there are studies that have shown that children with CHD have increased incidence of mood disorders (such as anxiety and fear), as well as delays in cognitive development, below average school performance, and poor social interactions [10, 13, 14].

As a national referral center for the treatment of CHD patients in Colombia, and recognizing the importance of an integral approach, the interest for the evaluation of QoL brought our institution to assemble a cohort of patients with Infrequent CHD (I-CHD), known as the PINOCCHIO Cohort. Through a research program sponsored by the Colombian government, we recruited patients with five I-CHD that are considered as neglected diseases in our country (Colombian Government: Procedural Resolution 2048 de 2015 and FECOER). In this manuscript, we describe QoL in children with I-CHD, both self-reported and through the perception of their caregivers. As a secondary objective, we describe changes in QoL after one-year of follow-up, both self-reported and through the perception of their caregivers.

## Methods

The PINOCCHIO cohort was assembled between August 2016 and September 2018 in a referral cardiovascular center in Bogotá, Colombia. Patients who fulfilled the following criteria were included: 1) age between 2 and 18 years; 2) confirmed diagnosis of one of the following selected I-CHD: Ebstein’s Anomaly (EA), Heterotaxy Syndrome (HTX), Interrupted Aortic Arch (IAA), Pulmonary Valve Stenosis (PVS) or Williams’ Syndrome (WS). Patients who were not feasible to be followed-up due to their place of residence or had a life expectancy of less than 6 months, were excluded.

After informed consent, a trained psychologist performed individualized QoL assessments for each child and their respective caregiver in separate moments, both at baseline and after one-year follow-up. All assessments were performed in the Congenital Heart Disease Institute at Fundación Cardioinfantil-Instituto de Cardiología in Bogotá.

### The pediatric quality of life inventory - PedsQL 4.0

For QoL evaluation the PedsQL 4.0 scale was used, (Spanish version) validated in Spanish by Vélez et al. [15], including generic and cardiac-specific modules for patients and their caregivers; this questionnaire has been used previously by other researchers for QoL analysis in CHD [7, 10]. The first module evaluates four domains of QoL: physical functioning (8 items), emotional functioning (5 items), social functioning (5 items) and school functioning (5 items). The cardiac-specific module evaluated seven different domains: cardiac symptoms (7 items), adherence to treatment (5 items; optional only if the patient is on pharmacologic treatment), perceived physical appearance (3 items); anxiety towards treatment (4 items); cognitive status (5 items) and communication skills (3 items). For children between 2 and 4 years of age only caregivers answered the questionnaire. For self-reported QoL, the questionnaire is divided by age: pre-school children (5–7 years of age), school children (8–12 years of age) and adolescents (13–18 years of age). The parent/caregiver proxy report is categorized equally for children between 2 and 18 years of age. A five-point Likert scale is used to score the questionnaire from 0 (never) to 4 (almost always). Scores are then transformed to a 0–100 scale where 0 = 100, 1 = 75, 2 = 50, 3 = 25 and 4 = 0. For children between 5 and 7 years-old the Likert scale is simplified to a 3 point scale as follows: 0 = never, 1 = sometimes and 2 = almost always [10, 15]. The questionnaire is usually completed in 10 to 15 min. Although there is no specific cut-off point, most authors have considered a score of less than 70 as a negative effect on QoL, and therefore this value was used for analysis [10, 16].

### Statistical analysis

Scores for general and cardiac-specific modules are presented as median and interquartile range. A Mann-Whitney U test was used to compare scores for general and cardiac modules between patients and caregivers. Comparisons within patients’ and caregivers’ baseline and one-year follow-up scores, were done using a Wilcoxon test. A *p* value of less than 0.05 was considered as statistically significant. SPSS v22 was used to conduct all statistical analysis.

## Results

**Fig. 1:**
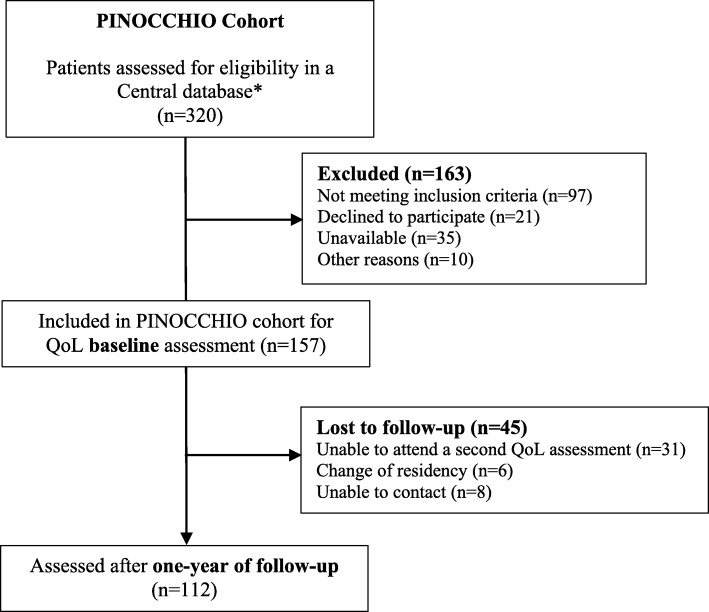
PINOCCHIO Cohort flow chart. *Central database was assembled with data from two main databases: 1) Congenital Heart Defects Institute, patients with congenital heart disease attending to this institute; 2) Cardiovascular and Percutaneous Interventions, patients attending to these medical services to receive treatments according to CHD diagnosis

At baseline, QoL assessment was achieved in 157 children included in the PINOCCHIO cohort; complete QoL evaluation at one-year follow-up was achieved in 112/157 patients (71%). Losses in follow-up mainly related with non-availability of caregivers for a second QoL assessment, change of address, and non-availability for contact (Figure 1 in Appendix). Demographic and clinical characteristics for patients at baseline (*n* = 157), patients with follow-up (*n* = 112), as well as for those lost to the follow-up (*n* = 45), are presented in Table 1. Age and sex distribution, I-CHD type, prenatal diagnosis, and previous surgical or percutaneous interventions, were very similar among the group. The most frequent I-CHD diagnosis was PVS (> 44%) followed by EA (> 35%); more than 10% of the children had a prenatal diagnosis, and more than 60% had a previous surgical or percutaneous intervention.

**Table 1:** Demographic and clinical patient’s characteristics by baseline, follow-up and lost to follow-up groups

	Baseline (*n* = 157)	Follow-up (*n* = 112)	Lost to follow-up (*n* = 45)
Age years, median (IQR)	6.0 (3.0–11.0)	7.0 (4.0–11.7)	6.0 (3.0–10.5)
Sex female, n (%)	85 (54.1)	62 (55.4)	23 (51.1)
I-CHD distribution, n (%)			
EA	56 (35.7)	40 (35.7)	16 (35.5)
HTX	9 (5.7)	4 (3.6)	5 (11.1)
IAA	15 (9.6)	11 (9.8)	4 (8.9)
PVS	71 (45.2)	51 (45.5)	20 (44.4)
WS	6 (3.8)	6 (5.4)	–
Prenatal diagnosis, n (%)	25 (15.9)	13 (11.6)	12 (26.7)
Previous surgical or percutaneous intervention, n (%)	100 (63.7)	74 (66.1)	26 (57.8)

*IQR* Interquartile range, *EA* Ebstein’s Anomaly, *HTX* Heterotaxy Syndrome, *IAA* Interrupted Aortic Arch, *PVS* Pulmonary Valve Stenosis, *WS* Williams’ Syndrome

Results for the PedsQL 4.0 general and cardiac-specific modules showed that patients had a better perception of their own QoL, when compared to their caregivers’ perception. Although there was a difference in scores both at baseline and at one-year follow-up, a statistically significant difference was only found for the cardiac-specific module (baseline *p* value = 0.019; follow-up *p* value = 0.034). For both patients and caregivers, scores were generally lower at follow-up. Results are presented in Table 2.

**Table 2:** Comparisons between patients and their caregivers on general and cardiac-specific total scores

Variable	Baseline			Follow-up		
	Patient (*n* = 85)	Caregiver (*n* = 112)	*p* value	Patient (*n* = 85)	Caregiver (*n* = 112)	*p* value
General-specific total score, median (IQR)	74.4 (64.1–80.4)	68.4 (59.6–83.7)	0.296	72.8 (59.2–85.9)	69.9 (58.1–83.7)	0.332
Cardiac-specific total score, median (IQR)	79.6 (69.7–87.4)	73.6 (62.6–84.3)	0.019	75.0 (67.1–87.1)	73.1 (59.5–83.8)	0.034

*IQR* Interquartile range

Results for each category of the general-specific module in patients showed that emotional health (baseline median = 65.0, IQR = 55.0–88.7; follow-up median = 70.0, IQR = 56.2–88.7) and school functioning (baseline median = 70.0, IQR = 55.0–80.0; follow-up median = 65.0, IQR = 50.0–85.0) are perceived as being more affected (scores < 70). However, there were no statistically significant differences between baseline and one-year follow-up scores in any of the categories of this module. With respect to the cardiac-specific module, cognitive status obtained the lowest score both at baseline and follow-up (median = 75.0, IQR = 55.0–95.0; median = 70.0, IQR = 45.0–90.0, respectively), along with cardiac symptoms (baseline median = 78.6, IQR = 60.7–98.3; follow-up median = 71.4, IQR = 60.7–85.7). There were no statistically significant differences in scores at baseline compared to one-year follow-up in any of the categories of the cardiac-specific module. Results for patients in all categories are presented in Table 3.

**Table 3:** General and cardiac-specific QoL scores assessed by Patients: Baseline versus Follow-up

	Baseline (*n* = 85)	Follow-up (*n* = 85)	*p* value
General-specific module, median (IQR)			
Physical health	75.0 (63.3–93.0)	78.1 (66.4–92.9)	0.906
Emotional health	65.0 (55.0–88.7)	70.0 (56.2–88.7)	0.850
Social functioning	80.0 (60.0–95.0)	80.0 (60.0–90.0)	0.224
School functioning	70.0 (55.0–80.0)	65.0 (50.0–85.0)	0.266
Total	74.4 (64.1–80.4)	72.8 (59.2–85.9)	0.496
Cardiac-specific module, median (IQR)			
Cardiac symptoms	78.6 (60.7–98.3)	71.4 (60.7–85.7)	0.161
Adherence to treatment	100 (85.0–100)	100 (93.7–100)	0.430
Anxiety towards treatment	82.1 (68.8–99.1)	85.7 (64.3–100)	0.242
Cognitive status	75.0 (55.0–95.0)	70.0 (45.0–90.0)	0.440
Communicative skills	83.3 (58.3–100)	91.7 (66.7–100)	0.739
Total	79.6 (69.7–87.4)	75.0 (67.1–87.1)	0.209

*IQR* Interquartile range

Results for caregivers in each category of the general-specific module showed that the most affected areas were physical health (baseline median = 75.0, IQR = 59.4–84.4; follow-up median = 75.1, IQR = 57.2–90.1), emotional health (baseline median = 65.0, IQR = 50.0–80.0; follow-up median = 65.0, IQR = 50.0–75.0) and school functioning (baseline median = 70.0, IQR = 50.0–85.0; follow-up median = 65.0 IQR = 41.2–85.0). However, there were no statistically significant differences between baseline and one-year follow-up scores in any of the categories of this module.

In the cardiac-specific module, caregivers perceived a bigger impact in most categories, but the lowest score was found for cognitive status (baseline median = 65.0, IQR = 45.0–80.0; follow-up median = 60.0, IQR = 40.0–80.0) and anxiety towards treatment (baseline median = 71.4, IQR = 60.7–89.3; follow-up median = 71.4, IQR = 54.5–85.7). Cardiac symptoms and communicative skills obtained borderline scores. There were no statistically significant differences between scores at baseline and one-year follow-up in any of the categories of the cardiac-specific module. Results for caregivers in all categories are presented in Table 4.

**Table 4:** General and cardiac-specific QoL scores assessed by Caregivers: Baseline versus Follow-up

	Baseline (*n* = 112)	Follow-up (*n* = 112)	*p* value
General-specific module, median (IQR)			
Physical health	75.0 (59.4–84.4)	75.1 (57.2–90.1)	0.975
Emotional health	65.0 (50.0–80.0)	65.0 (50.0–75.0)	0.668
Social functioning	80.0 (60.0–95.0)	75.0 (60.0–90.0)	0.143
School functioning	70.0 (50.0–85.0)	65.0 (41.2–85.0)	0.631
Total	68.4 (59.6–83.7)	69.9 (58.1–83.7)	0.583
Cardiac-specific module, median (IQR)			
Cardiac symptoms	73.2 (57.1–85.7)	75.0 (57.1–85.7)	0.922
Adherence to treatment	100.0 (90.0–100)	100.0 (100–100)	0.116
Anxiety towards treatment	71.4 (60.7–89.3)	71.4 (54.5–85.7)	0.311
Cognitive status	65.0 (45.0–80.0)	60.0 (40.0–80.0)	0.499
Communicative skills	75.0 (50.0–100)	83.0 (50.0–100)	0.203
Total	73.6 (62.6–84.3)	73.1 (59.5–83.8)	0.545

*IQR* Interquartile range

## Discussion

In this study we found a lower perception of QoL in children with five different diagnoses of I-CHD, in the general as well as the cardiac modules of the PedsQL 4.0. Our findings also show that when caregivers are asked to evaluate their child’s QoL, scores were lower than those reported by the children themselves. These perceptions were maintained after one-year of follow-up, and in addition the scores were lower for both groups (children and caregivers).

The differences between caregivers and children in QoL assessments observed in the present study are consistent with other publications [7, 10, 11]. Ruggiero et al. showed that children may have a reasonable perception of QoL, while their parents reported lower scores, especially in older children and in those with more severe disease [10].

Our results regarding perception of QoL in specific categories of the questionnaire, such as emotional health and school functioning, support previous publications that showed than in children 3–11 years of age, the same areas are identified by patients as the most compromised [11]. For caregivers, the results are consistent for these same domains. However, perception related to the severity of the CHD varies. As shown in previous studies, severe heart conditions tend to have lower scores in specific domains and in general modules for both patients and caregivers [9, 11].

The difference in QoL perception might be associated with the patient’s own expectations compared to those of their parents or caregivers regarding their social, cognitive and intellectual abilities [10, 11, 17]. Thus, a caregiver’s assessment can be an essential aspect in the evaluation of a child with CHD from the perspective of the use of healthcare facilities, such as emergency department visits and office visits. Understanding this can enhance the communication between patients and their caregivers, and should help patients improve outcomes over time [7, 10]. As healthcare providers and members of a heart team, these and previous results suggest that it is necessary to monitor treatment decisions in the context of the social, emotional and cognitive expectations of children themselves in addition to their own perceptions [18]. It may be debatable to consider a QoL evaluation from a pediatric patient, nonetheless the questionnaires allow adequate data extraction from the child regarding their own perceptions to what they think their quality of life should be like. In addition to all the clinical information that is gathered when evaluating a child with CHD, many times as clinicians we tend to vision treatment protocols or interventions towards the best QoL possible, but these results as well as others previously cited, compels the clinician to factor in the patients’ expectations into the clinical decision-making process.

The strengths of this study are mainly related with the inclusion of a wide spectrum of I-CHD, from structural to syndromic, that currently have limited evidence regarding QoL assessments, since most of the research has focused on clinical outcomes. Our population represent a wide demographic and clinical spectrum in age, previous treatment and prenatal diagnosis as well as different socioeconomic and cultural backgrounds and diversity in educational level of the parents, which enriches both the content and the meaning of the results provided. However, this wide age spectrum may make it harder to detect the impact on QoL of different levels of development, social, and physical aspects.

Limitations of this study are mainly related to time of inclusion and with follow-up. First, the inclusion of children into the study was not done at a specific point in their diseases, and it is not strictly linked to a clinical or growth milestone at baseline or at follow-up. Thus, the changes seen over 1 year can be influenced by multiple factors, which individually or combined, can modify QoL due to clinical, schooling, or variables that were not measured in this study. Secondly, loss to follow-up might be associated with selection biases. Even though we characterized at baseline those patients lost to follow-up (Table 1) and did not find relevant differences when compared with those followed, un-measured variables such as new interventions, new clinical conditions, hospitalizations, or school or personal changes might impact QoL perceptions.

The integral and global evaluation of patients with CHD is gaining increasing clinical value in the recent medical literature. Our results help close this gap, and also confirm previous results with similar methodology that have examined QoL in patients with CHD and from both the patient and their caregivers perspective [7, 10, 16, 17]. Finally, these findings suggest that besides a thorough clinical evaluation, QoL assessment should be an integral part of the medical management of a patient with I-CHD.

## Conclusions

Children with I-CHD perceive their QoL as being compromised in different aspects of life. Assessment of QoL through their caregivers confirm these findings; however, children QoL scores were higher than scores assigned by caregivers. These perceptions were maintained after one-year of follow-up, when the scores were lower in both groups (children and caregivers). Based on these findings, we suggest that to provide high-quality care, besides a thorough clinical evaluation, QoL directly elicited by the child should be an essential aspect in the integral management of I-CHD and in the clinical decision-making process.

## Data Availability

The datasets used during the current study are available from the corresponding author.

## Appendix

### QoL scores for patients and their caregivers at follow-up, according to specific I-CHD

QoL perception according to the specific diagnosis showed that patients with Interrupted Aortic Arch (IAA) perceived a greater compromise, especially in the general module (median=67.4, IQR=61.9-70.6). Scores obtained for the cardiac-specific module were borderline (median=73.9, IQR=70.3-79.8). Although QoL total scores were lower for the general module (median=63.0, IQR=50.0-71.4), patients and caregivers perceived also a compromise in the cardiac-specific module (median=73.1, IQR=59.3-78.4). Patients with WS had the most divergent scores between patients and caregivers; perception of patients was more positive in the general (median=83.1, IQR=55.7-100) and cardiac-specific (median=86.4, IQR=61.2-100) modules than those by their caregivers (*general* median=62.4, IQR=56.8-77.9; *cardiac-specific* median=63.4, IQR=47.5-77.3). Results for general and cardiac modules according to I-CHD are presented in table 5.

**Table 5:** QoL scores for patients and their caregivers at follow-up, according to specific I-CHD

Diagnosis	General-specific section total score, median (IQR)		Cardiac-specific section total score, median (IQR)	
	Patient (*n* = 85)	Caregiver (*n* = 112)	Patient (*n* = 85)	Caregiver (*n* = 112)
EA	73.9 (58.7-84.8)	68.2 (53.5-81.8)	77.8 (71.3-89.8)	71.3 (58.9-82.7)
HTX	83.7 (83.7-83.7)	64.0 (53.7-68.4)	80.5 (80.5-80.5)	68.6 (61.4-69.8)
IAA	67.4 (61.9-70.6)	63.0 (50.0-71.4)	73.9 (70.3-79.8)	73.1 (59.3-78.4)
PVS	73.9 (57.0-87.5)	77.2 (64.1-86.9)	75.0 (62.0-88.6)	75.9 (60.2-85.2)
WS	83.1 (55.7-100)	62.4 (56.8-77.9)	86.4 (61.2-100)	63.4 (47.5-77.3)

*IQR* Interquartile range, *EA* Ebstein’s Anomaly, *HTX* Heterotaxy Syndrome, *IAA* Interrupted Aortic Arch, *PVS* Pulmonary Valve Stenosis, *WS* Williams’ Syndrome

## Abbreviations

CHD: Congenital heart defects
EA: Ebstein’s anomaly
HTX: Heterotaxy syndrome
IAA: Interrupted aortic arch
I-CHD: Infrequent congenital heart defects
IQR: Interquartile range
PROs: Patient-related outcomes
PVS: Pulmonary valve stenosis
QoL: Quality of life
WS: Williams’ syndrome
